# Individual Perceptions of the Efficacy, Quality, and Safety of Sexual Health Care Delivered via a Direct-to-Consumer Telehealth Platform: Retrospective Analysis of Data From a Cross-Sectional Survey Study

**DOI:** 10.2196/95561

**Published:** 2026-09-15

**Authors:** Michael Bohl, Lauren Governale, Peter Stahl, Knox Beasley, Jessica Yu

**Affiliations:** 1 Hims & Hers Health, Inc, 2269 Chestnut Street, Suite 523, San Francisco, CA, 94123, United States, 1 6508048434; 2 You Health Medical Groups, San Francisco, CA, United States

**Keywords:** telehealth, erectile dysfunction, premature ejaculation, sexual dysfunction, sexual health, quality, safety

## Abstract

**Background:**

Increasingly, individuals are seeking treatment for sexual health concerns through direct-to-consumer (DTC) telehealth platforms, and there is ongoing debate about whether such platforms can deliver safe and effective care.

**Objective:**

The objective of this study was to examine how men who sought sexual health treatment through a large national DTC telehealth platform viewed the quality and safety of their care, and how these perceptions differed from those of men who sought other types of treatment through the platform.

**Methods:**

We conducted a retrospective analysis of data gathered from an online survey sent to individuals who accessed treatment through the DTC telehealth platform. Individuals completed the survey between June 30, 2025, and July 3, 2025. The survey consisted of 4 sets of items that asked respondents about their perceptions of the quality and safety of the care they accessed. We used descriptive statistics to summarize individuals’ responses to survey items and chi-square tests to compare the responses of those who accessed sexual health treatment with those who accessed other types of treatment.

**Results:**

The survey was completed by 497 men who accessed sexual health treatment and 512 men who accessed other types of treatment through the platform. Among sexual health participants, 397 of 497 (≥80%) reported favorable impressions of the quality and safety of care across most survey items. Sexual health participants were more likely than nonsexual health participants to view the online intake as similar to an in-person appointment (*χ*²_1_=12.4; *P*<.001) and more likely to rate the telehealth experience as better than in-person care with respect to privacy and discretion (*χ*²_1_=14.8; *P*<.001); the convenience of managing their care (*χ*²_1_=14.9; *P*<.001); the thoroughness of questions asked during the intake (*χ*²_1_=23.5; *P*<.001); the clarity of treatment options and information (*χ*²_1_=17.1; *P*<.001); the overall sense of safety and medical quality (*χ*²_1_=18.2; *P*<.001); their trust in the care model and medical providers (*χ*²_1_=18.2; *P*<.001); and the transparency about possible side effects and risks (*χ*²_1_=12.8; *P*<.001).

**Conclusions:**

Men who accessed sexual health treatment via a national DTC telehealth platform had positive perceptions of the overall quality and safety of care. Their responses to specific survey items may inform future research on the efficacy, quality, and safety of DTC telehealth, as well as product and service development in virtual health care.

## Introduction

The growth of telehealth as a means of accessing care during and following the COVID-19 pandemic is well documented [1]. One application of telehealth—direct-to-consumer (DTC) platforms that offer access to sexual health care for men seeking treatment for concerns such as erectile dysfunction (ED)—has become increasingly popular in recent years.

One study examining online traffic to 6 major DTC telehealth platforms offering treatment for ED reported a 1688% increase in unique quarterly visitors between the fourth quarter of 2017 and the fourth quarter of 2019 [2]. This increase in platform traffic occurred despite otherwise stable online search volume for the term “erectile dysfunction.” Other research from 2020 found that 1 of the 3 most common health conditions for which patients used DTC telehealth was ED [3]. This accounted for 21.1% of visits, compared with less than 2.3% of in-person primary care visits (although the authors noted that their results were limited by the set of services offered by the DTC telehealth company).

Paralleling the increased uptake of men’s sexual health care through DTC telehealth is the increased focus on the question of whether such care can be delivered effectively and safely. In one study that analyzed the quality of patient information about ED across 5 men’s telehealth platforms, researchers found that information quality scores were highest for the larger platforms, but overall concluded that men seeking online ED care were at risk of receiving incorrect information [4]. In a separate retrospective analysis of men aged 40 years and under, investigators noted that certain pathologies (including diabetes, dyslipidemia, hypogonadism, varicocele, and abnormal semen analysis) were discovered during in-person visits and asserted that these would be missed by the screening questionnaires used by DTC telehealth platforms—implying that DTC telehealth platforms provide substandard care [5]. Another study also found that platforms had low adherence to American Urological Association (AUA) guidelines for treating ED [6], although telehealth-specific guidelines do not exist, and the study did not include a comparison with in-person care [7].

The work detailed above has been conducted by expert clinicians and researchers, yet it lacks the perspective of consumers who actively engage with platforms. It is important to understand how such individuals view the care they receive and to combine these insights with the perspectives of clinical, research, and other professional stakeholders involved in the design and development of DTC telehealth platforms. Doing so can ultimately ensure that such platforms are accessible, effective, customer- and patient-friendly, and safe.

To that end, this study builds on previous work by our team in which we conducted a retrospective analysis of data collected from customers who accessed care via a national DTC telehealth platform and completed a survey exploring their attitudes, beliefs, and opinions about the quality and safety of the care they received. Our findings indicated that the majority of individuals who engaged with the platform and completed the survey had positive perceptions of the efficacy, quality, and safety of the care they received, and viewed the experience as good as or better than prior in-person health care experiences [8]. In this study, we focus specifically on men who sought sexual health treatment through the platform to (1) gain a better understanding of how men who access sexual health care via DTC telehealth platforms perceive the quality and safety of their care and (2) determine whether there are differences in perceptions of quality and safety between men who seek sexual health care and those who seek other types of care.

## Methods

### Study Design

This study is a follow-up to and extension of data we previously reported [8]. In the original study, we conducted a retrospective analysis of data gathered from an online survey, originally administered for quality improvement purposes, to better understand individuals’ attitudes about the quality and safety of care they accessed via a large national DTC telehealth platform. In this study, we conducted a retrospective analysis of data gathered specifically from men who accessed sexual health services and compared their responses to those of men who accessed other types of treatment.

### Telehealth Platform

Hims & Hers is a DTC telehealth platform available to adults seeking treatment related to sexual health, dermatology, mental health, weight management, and more. Individuals access the platform via Hims [9], intended for a male audience, or Hers [10], intended for a female audience, and select the health care concern for which they would like to receive treatment. Individuals are directed to complete a comprehensive intake form asking about their symptoms, medical history, medications, and treatment preferences. A medical provider licensed in the individual’s state then thoroughly reviews the information, following up with the individual as needed to ask any additional questions that could inform the provider’s assessment and treatment decision. All licensed medical professionals providing care on the platform are employed by or contracted with You Health, a professional corporation owned and operated by licensed health care providers. The providers make independent clinical determinations as to whether treatment is appropriate for each individual and communicate that decision to them, sharing a diagnosis and treatment plan if appropriate. If medication is prescribed, individuals sign up for a subscription to receive it from a licensed pharmacy at predetermined intervals. The subscription also includes access to a mobile app, in which the individual can find additional education about their conditions and treatments and in which unlimited messaging with the individual’s care team is available.

### Participants

Participants were men aged 18 years and older who accessed care through the Hims & Hers DTC telehealth platform, responded to an online survey about their experience, and had an active subscription at the time of survey completion. Participants were divided into 2 cohorts. “Sexual health participants” were defined as men who self-identified, via a survey question, as using treatments or products from Hims & Hers to treat a sexual health concern such as ED or premature ejaculation. “Nonsexual health participants” were men who self-identified as using treatments from Hims & Hers to treat concerns related to overweight or obesity, hair loss, mental health, skin care, or other conditions.

### Ethical Considerations

The survey used in this study was originally part of a quality improvement initiative at Hims & Hers. This initiative did not undergo institutional review board approval; however, all participants were required to review an electronic consent form prior to beginning the survey. The consent form informed participants that participation in the survey was voluntary and would not affect their treatment on the platform, that their responses would be confidential, and that the resulting data would be used to inform improvements to the platform—including its products, services, and user experience. After reviewing the form, individuals electronically signed it to indicate their understanding of these terms. Survey completers were entered into a raffle to win one of three US $100 Visa gift cards.

This study is a retrospective analysis of data collected during the quality improvement initiative. All data were deidentified prior to inclusion in the analysis. This study was approved by the WCG Institutional Review Board (Protocol 001, approval: 1-1913411-1) on October 30, 2025. As a retrospective analysis of deidentified data, the study received a waiver of informed consent, and no additional informed consent was required. Participants did not receive additional compensation for this study.

### Survey

The survey was originally developed by the Hims & Hers Customer Insights and User Experience Research team for the purpose of product and quality improvement; the deidentified data were then analyzed for this research. Prior to writing the survey, the team consulted with clinical, product, and quality experts across the organization to determine topics of interest. Based upon this input, the team then developed specific questions related to customers’ overall experience with the telehealth platform, their awareness of safety practices used by the platform, their experience with the online intake, their sentiments toward the support available through the platform, their experience with the telehealth platform compared with prior in-person care experiences, their experience with the telehealth platform compared with other telehealth experiences, and their feedback on future product development. These questions were reviewed by the aforementioned clinical, product, and quality experts to ensure consistency with the desired topics. Following their approval, the survey items were reviewed by 2 lead researchers (LG and JY) for clarity and readability. The survey was then programmed into Qualtrics, an online survey platform compliant with the Health Insurance Portability and Accountability Act of 1996, after which it underwent quality assurance prior to being made available to participants. The team conducted a logical flow test to ensure that any skip logic and branching within the survey performed as planned and that the survey was available for completion on participants’ desktop or mobile devices, as well as on different internet browsers. Finally, the team sent a test survey to a small group of respondents to ensure that survey completion and data collection went as expected. No revisions were made or required following this test.

The final survey had an estimated time to completion of 15 to 20 minutes and comprised 4 sets of items deemed relevant for this analysis. Six items were related to individuals’ overall perceptions of quality and safety on the platform. Responses were captured on a 5-point Likert scale, with 1 being “strongly disagree” and 5 being “strongly agree.” Five items were related to their experiences with the online intake. Responses were captured on a 5-point Likert scale, with 1 being “strongly disagree” and 5 being “strongly agree.” Five items were related to individuals’ awareness of safety practices used by the platform. Responses were captured on a 5-point Likert scale, with 1 being “not at all aware” and 5 being “very aware.” Ten items asked participants to compare their experiences on the telehealth platform to prior in-person health care experiences. Responses were captured on a 5-point Likert scale, with 1 being “much worse” and 5 being “much better.” A detailed summary of these questions is included in Multimedia Appendix 1.

A link to the online survey was sent via email to a random sample of 280,733 customers with an active subscription to the Hims & Hers platform between June 30, 2025, and July 3, 2025. This recruitment sample size was determined based upon the following: (1) the desire to have a total of 2000 customers accessing various types of treatment through the platform complete the survey, with 2000 respondents considered robust in consumer and user experience research [11,12], and (2) an anticipated survey completion rate of 1% based upon historical quality improvement and user experience work within the company. Participants were required to complete the entire survey to be included in this analysis, except for questions that asked them to compare their telehealth experience to prior in-person care experiences. This was done to consider the possibility that some participants may not have had recent in-person care experiences.

### Statistical Analysis

For items asking participants to rate their agreement with specific statements related to their experience of the quality and safety of care available through the platform, we dichotomized responses such that responses of 1 to 3 were coded as “not agree” and 4 to 5 were coded as “agree.” For items asking participants to rate their agreement with specific statements related to their experience with the online clinical intake, we again dichotomized responses such that those of 1 to 3 were coded as “not agree” and 4 to 5 were coded as “agree.” For items asking participants to rate their awareness of specific safety practices used by the platform, we coded responses of 1 to 3 as “not aware” and 4 to 5 as “aware.” Finally, for items asking participants to compare their experience on the telehealth platform to other in-person health care experiences, we trichotomized variables such that responses of 1 to 2 were coded as “worse,” 3 was coded as “the same,” and 4 to 5 were coded as “better.” We did so for 2 reasons. First, across items, the distribution of participant responses was heavily clustered at higher values. Second, this was the approach used in our previously published work [8], thus allowing for consistency and better comparison between the 2 studies.

We then performed descriptive statistics in Microsoft Excel (version 16.107.2) to report available baseline data and the number and percentage of participants who responded to survey items. We used chi-square tests of independence to examine the relationship between treatment accessed and perceptions of quality and safety. We considered the use of chi-square tests to be appropriate given the way in which we collapsed participant responses, as well as our objective of understanding whether being a sexual health or nonsexual health participant was associated with a greater likelihood of a favorable perception of the quality and safety of care available through the platform. To account for an increase in the familywise error rate due to multiple, simultaneous testing, we used a Bonferroni correction [13] and divided the significance level of *P*<.05 by the number of tests (in our study, 26) for a Bonferroni-adjusted significance level of *P*<.002.

For the subset of items asking participants to compare their telehealth experiences to prior in-person health care experiences, we conducted additional 2×2 post hoc comparisons for 8 of the initial 2×3 tests that had found statistically significant differences to determine how the groups differed in their responses. Given that the original omnibus Bonferroni correction resulted in a highly stringent and conservative significance level of *P*<.002, we adopted this significance level rather than use an additional correction that might have been overly strict.

## Results

### Overview

A total of 1009 men who were active customers on the Hims & Hers telehealth platform at the time of survey completion responded to the survey. Of these 1009 men, 497 (49%) were receiving treatment for a sexual health concern, specifically ED or premature ejaculation, and are hereafter referred to as “sexual health participants.” The remaining 512 (51%) men were receiving treatment for nonsexual health concerns and are hereafter referred to as “nonsexual health participants.” Of the 512 nonsexual health participants, 231 (45%) were receiving treatment for hair loss, 217 (42%) were receiving treatment for weight loss, 17 (3%) were receiving treatment for mental health concerns, 4 (1%) were receiving treatment for skin-related concerns, 41 (8%) were receiving treatment for more than one nonsexual health concern, and 2 (<1%) were receiving other nonsexual health treatment available through the platform.

### Overall Experience

Overall, sexual health participants appeared to have a positive experience with the telehealth platform. Eighty percent (412/497) or more stated that they trusted the telehealth platform to offer safe and effective care, viewed the entire process—from intake to prescription—as medically safe, fully understood the risks and benefits of their prescribed medication, were confident that their prescribed medication was safe and appropriate for them, believed that the telehealth platform followed the proper rules and safety standards for health care, and felt that the provider they interacted with asked enough questions to fully understand their health history.

Table 1 summarizes the results of the chi-square tests comparing sexual health and nonsexual health participants with regard to overall experience with the telehealth platform. These tests found no statistically significant differences between the 2 groups at the Bonferroni-corrected significance threshold with respect to the percentage of participants who reported agreement with the items.

**Table 1.** Participants’ overall perceptions of quality and safety on the telehealth platform.

Survey item: To what extent do you agree with the following statements about your experience with the telehealth platform?	Sexual health participants^a^ (n=497), n (%)	Nonsexual health participants^a^ (n=512), n (%)	Chi-square (*df*)	*P* value
I was confident my prescription was safe and appropriate for me.	416 (84)	423 (83)	0.2 (1)	.65
The entire process–from intake to prescription–felt medically safe.	415 (84)	412 (80)	1.6 (1)	.21
I trust the telehealth platform to offer safe and effective care.	419 (84)	398 (78)	7.1 (1)	.01
I fully understood the risks and benefits of the medication.	418 (84)	419 (82)	0.9 (1)	.34
I believe the telehealth platform follows the proper rules and safety standards for health care.	414 (83)	406 (79)	2.6 (1)	.10
I felt the provider asked enough questions to fully understand my health history.	397 (80)	369 (72)	8.4 (1)	.004

a Responses indicate agreement (“agree” or “strongly agree”).

### Intake Form

Sexual health participants’ responses to a series of questions about their experience with the telehealth platform’s online intake process indicated that they had favorable impressions of the process. Eighty-five percent (424/497) or more of participants stated that the intake form felt easy to complete, offered a more efficient and less stressful experience than an in-person visit, was similar to the types of questions a provider might ask during an in-person appointment, made them feel more confident in starting treatment, and clearly explained available treatment options.

Results of the chi-square tests comparing the 2 groups with regard to their experiences with the online intake process indicated a statistically significant difference in the percentage of participants who felt that the online intake was similar to the types of questions they might encounter from a provider during an in-person appointment. Sexual health participants appeared to report a similarity more often (*χ*²_1_=12.4; *P*<.001) (Table 2).

**Table 2.** Participants’ perceptions of the online intake process.

Survey item: When you first became a customer you filled out an intake form as part of that process. Thinking back to that initial experience, how would you rate the intake experience with the telehealth platform?	Sexual health participants^a^ (n=497), n (%)	Nonsexual health participants^a^ (n=512), n (%)	Chi-square (*df*)	*P* value
It felt easy to complete.	446 (90)	455 (89)	0.2 (1)	.65
It offered a more efficient and less stressful experience than an in-person visit.	436 (88)	430 (84)	2.9 (1)	.09
It made me feel more confident in starting treatment.	433 (87)	413 (81)	7.8 (1)	.005
It felt similar to the types of questions a provider might ask during an in-person appointment.	432 (87)	402 (79)	12.4 (1)	<.001
It clearly explained my treatment options.	424 (85)	410 (80)	4.8 (1)	.03

a Responses indicate agreement (“agree” or “strongly agree”).

### Awareness of Safety Practices

Table 3 summarizes sexual-health participants’ responses to a series of questions about their awareness of various safety practices implemented on the telehealth platform. About 61% (304/497) to 79% (391/497) of participants were aware of specific practices. Results of the chi-square tests comparing the 2 groups on their awareness of safety practices found no significant differences in responses at the Bonferroni-corrected significance threshold.

**Table 3.** Participants’ awareness of quality and safety practices used by the platform.

Survey item: How aware are you of the following safety practices used by the telehealth platform?	Sexual health participants^a^ (n=497), n (%)	Nonsexual health participants^a^ (n=512), n (%)	Chi-square (*df*)	*P* value
24/7 access to licensed providers for safety follow-up and questions	391 (79)	366 (71)	7.0 (1)	.008
Licensed pharmacists formulating compounded treatments	382 (77)	350 (68)	9.2 (1)	.002
Medications sourced from FDA-regulated^b^ facilities	350 (70)	336 (65)	2.7 (1)	.10
Ongoing provider training and chart review	311 (63)	279 (54)	6.8 (1)	.009
Pharmacy safety protocols (eg, clean rooms and sterility checks)	304 (61)	273 (53)	6.3 (1)	.01

a Responses indicate agreement (“aware” or “very aware”).

b FDA: US Food and Drug Administration.

### Comparison With Traditional In-Person Health Care

**Figure 1.**
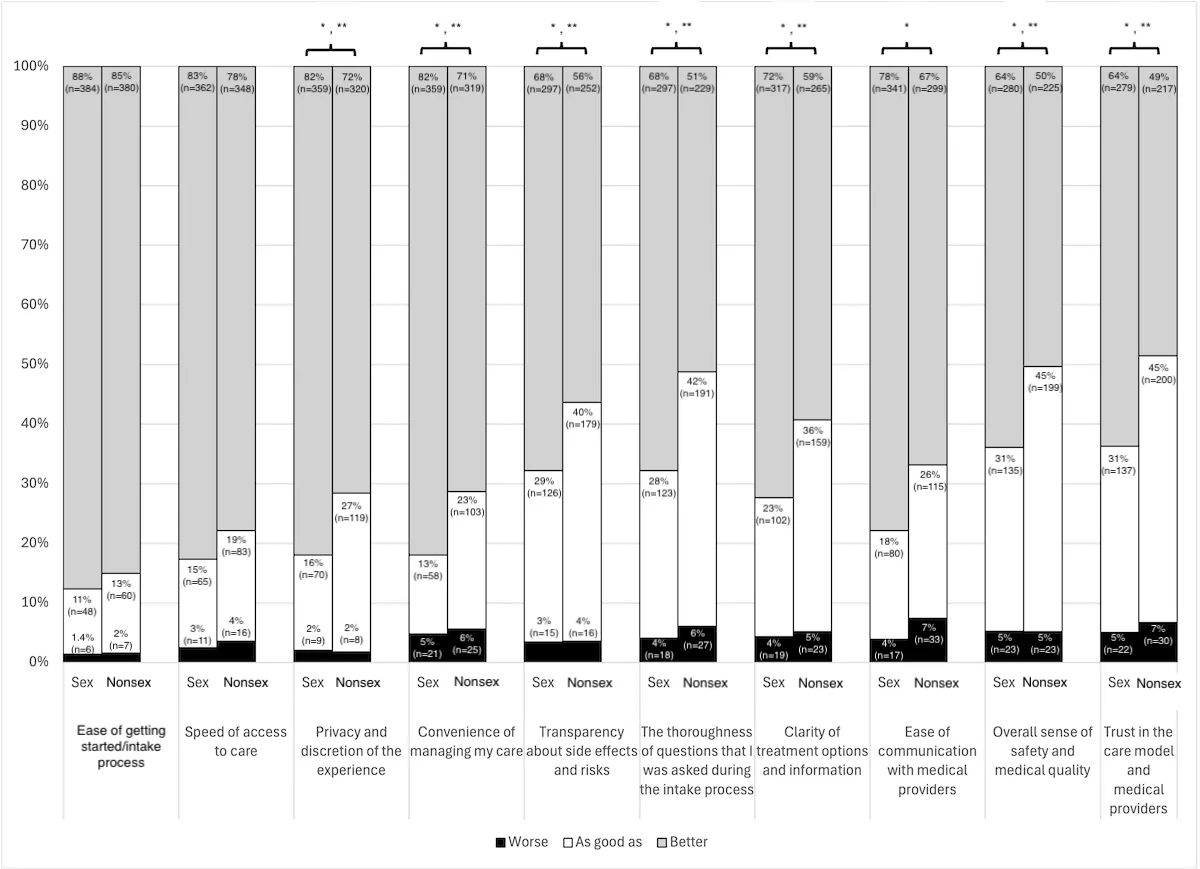
Participants’ comparisons of their telehealth experiences to prior in-person health care experiences, sexual health participants (“Sex”; n=438) vs nonsexual health participants (“Nonsex”; n=447). Comparisons denoted with “*” were 2x3 tests that were significant at the Bonferroni-adjusted significance level of *P*<.002. Comparisons denoted with “**” were post hoc pairwise tests that were significant at the Bonferroni-adjusted significance level of *P*<.002.

Among the respondents, 438 sexual health participants and 447 nonsexual health participants completed a series of items comparing their telehealth experience with prior in-person health care experiences. Figure 1 visualizes their responses. Ninety-five percent (416/438) or more of sexual health participants viewed each assessed aspect of the telehealth experience as at least as good as prior in-person health care experiences, and 64% (280/438) or more viewed each aspect as superior to prior in-person health care experiences. Results of the chi-square tests comparing the responses of sexual health and nonsexual health participants showed statistically significant differences between the 2 groups for 8 items. Post hoc comparisons found statistically significant differences between the 2 groups for 7 items. Specifically, sexual health participants appeared to report more often that their telehealth experience was *better* than in-person care, and nonsexual health participants appeared to report more often that their telehealth experience was *as good as* in-person care, with respect to the privacy and discretion of the experience (*χ*²_1_=14.8; *P*<.001); the convenience of managing their care (*χ*²_1_=14.9; *P*<.001); the thoroughness of questions they were asked during the intake process (*χ*²_1_=23.5; *P*<.001); the clarity of treatment options and information (*χ*²_1_=17.1; *P*<.001); the overall sense of safety and medical quality (*χ*²_1_=18.2; *P*<.001); their trust in the care model and medical providers (*χ*²_1_=18.2; *P*<.001); and the transparency about possible side effects and risks (*χ*²_1_=12.8; *P*<.001).

## Discussion

### Summary of Findings

In this study, we examined how men who accessed sexual health care via a DTC telehealth platform responded to a survey regarding their perceptions of the quality and safety of care they received and compared their responses to those of men who accessed other types of care through the platform. We found that across survey items, the majority of both sexual health and nonsexual health participants had favorable perceptions of the quality and safety of care accessible via the platform, with few statistically significant differences between groups. Overwhelmingly, both sexual health and nonsexual health participants indicated agreement with items that assessed their overall perceptions of quality and safety, such as their belief that the process of completing an intake and receiving a prescription felt medically safe and their trust in the telehealth platform to offer safe and effective care. They also indicated agreement with items that assessed their experience with the online intake process, such as the intake thoroughly explaining treatment options and helping them feel more confident in starting treatment. However, sexual health participants more often reported that the online intake felt similar to the type of questioning they might experience during an in-person appointment. Both sexual health and nonsexual health participants had variable awareness of safety practices used by the telehealth platform. Participants most often reported knowing that they had 24/7 access to providers and that licensed pharmacists formulated the compounded treatments available through the platform; they least often reported knowing about ongoing provider training and pharmacy safety protocols. Notably, both sexual health and nonsexual health participants rated the telehealth experience as being at least as good as prior in-person care experiences, though sexual health participants more often reported the telehealth experience as being superior to in-person care, and nonsexual health participants more often reported the telehealth experience as being as good as in-person care, with respect to several items in this domain.

### Interpretation of Findings

Previous research on individuals’ experiences with DTC telehealth indicates that individuals generally report high satisfaction with such services [14,15] and appreciate their ease of use [15]. However, few studies have focused specifically on individuals’ perceptions of the quality and safety of care available through such services, and those that have present somewhat mixed findings. In one recent study, 151 Australian adults who had accessed DTC telehealth services completed a survey regarding several aspects of their care, including their reasons for using DTC telehealth and their perceived benefits and concerns. Results indicated that participants had positive sentiments toward their experiences—they felt “more in control” and “in charge” of their health concerns and believed they had “more correct knowledge” and were “better informed” through their DTC experience [16]. In another recent study, researchers conducted focus groups with Australian adults who had engaged with DTC electronic prescription services and found that participants had varied feedback about their experiences. While participants believed such services addressed several gaps in health care delivery, including access, they also appeared to have some concerns about the overall quality and safety of these services due to the perceived need for adequate health literacy to navigate such platforms, limited communication between consumers and prescribers, and the potential for overprescribing [17].

Findings from our study are consistent with, and extend, the existing literature. Both sexual health and nonsexual health participants had overall favorable perceptions of the quality and safety of care available through the platform. However, there were certain areas in which the responses of sexual health participants appeared to deviate from those of nonsexual health participants. These areas were related to the accessibility of the telehealth experience, the online intake process, and the overall care model. Sexual health participants more often viewed the convenience, privacy, and discretion of the telehealth experience as being better than that of in-person care. They also more often viewed the online intake as being similar to the type of questioning they might encounter during an in-person visit, more often rated the thoroughness of the online intake as being better than that of in-person care, and more often rated the clarity of treatment options and information and transparency about side effects and risks presented during the telehealth experience as being better than that of in-person care. Finally, sexual health participants more often rated the overall sense of medical safety and quality on the telehealth platform and their trust in the care model and providers on the platform as being better than that of in-person care.

One explanation for the above may be the maturity of the sexual health care offering compared to other care offerings on this particular telehealth platform. Sexual health care was the platform’s original focus. With that, many aspects of the sexual health consumer and patient experience—including the online intake, the education and information provided on the platform’s website, the multimedia content available via the platform’s mobile app, and the way in which customer insights and user research are used for product and service delivery—have had more time to develop and improve. Thus, the sexual health offering may provide an unintentionally enhanced experience compared to its less mature counterparts, which translates into individuals’ perceptions of care.

Findings from our study also indicate that while individuals may have positive perceptions of the quality and safety of care they receive through DTC telehealth platforms, they may be unclear about specific quality and safety practices that are implemented on such platforms. This disconnect can help explain what quality- and safety-related factors are most important to consumers as well as how to educate consumers on important quality and safety considerations to take into account when choosing to engage with DTC telehealth.

Our findings are notable given the existing literature on the quality and safety of sexual health care delivered via DTC telehealth. As previously mentioned, there are currently no telehealth-specific guidelines for treating ED. However, a 2024 white paper jointly published by the AUA and the Sexual Medicine Society of North America begins to move the conversation in that direction [18]. The paper proposes a hybrid model of care that takes advantage of telehealth for certain parts of the diagnostic and treatment plan while still anchoring components of the full care journey to in-person care. This is a welcome recognition of telehealth’s increased uptake and the roles it can play. At the same time, the white paper, by its nature, does not update formal clinical practice guidelines to give clear guidance on how and when DTC telehealth can be used for men’s sexual health treatment. As the body of literature continues to grow and shed light on different stakeholders’ perceptions of telehealth’s quality and safety, including those of consumers as in this study, professional organizations should build on existing frameworks and update guidelines accordingly to reflect current practices.

### Limitations

Our study has limitations. One is potential sampling bias. Only individuals who were active customers of the telehealth platform were invited to complete the survey, and such individuals may have experienced more positive outcomes or been more satisfied with the telehealth platform than former customers. Relatedly, the active customers who responded to the survey may have had stronger feelings (positive or negative) about telehealth that they wanted to share than those who chose not to complete the survey. Another limitation is the use of a survey that was developed internally and was not a validated instrument. While the survey was designed using methodology consistent with best practices in user experience research, it was not tested for construct validity or internal consistency. In addition, the phrasing of the set of questions within the survey asking participants to compare their telehealth experience with prior in-person health care experiences may have been open to interpretation. The question asked participants to consider “traditional health care [they had] used.” As written, participants may have considered any number of health care–related interactions, some of which may not have provided a fair comparison. A third limitation is potentially limited generalizability, as the survey was specifically about the Hims & Hers telehealth platform. A final limitation is the study’s definition of sexual health medicine. Included in this study were males receiving care for ED and premature ejaculation, but other aspects of sexual health medicine—such as herpes, hypogonadism, and sexually transmitted infection treatment—were not examined. Strengths of the study include the large sample size and number of questions asked.

### Conclusions

It is clear that sexual health medicine has become a sought-after specialty in the DTC telehealth space. In addition to its convenience and accessibility, DTC telehealth is discreet. Stigma surrounds many sexual health issues, and patients may feel embarrassed, worried that they might be dismissed, or fearful about others finding out about their condition [19]. The privacy offered by DTC telehealth platforms, therefore, makes them an approachable venue for such care. Indeed, within the overall favorable perceptions participants had about the quality and safety of care available through these platforms, we found that 82% of sexual health participants viewed the privacy of telehealth platforms as better than in-person care, suggesting that telehealth is a popular venue for sexual health care because patients value this discretion.

Although there were few significant differences between the sexual health cohort and nonsexual health cohort, they are notable as they pertain to the online intake process and the provision of treatment information. The results open the door to future research analyzing why these differences exist and what lessons can be taken from sexual health—one of the earliest fields of medicine to be part of the DTC telehealth boom—and applied to the ever-growing list of medical specialties now using that modality.

## Supplementary material

10.2196/95561 Multimedia Appendix 1 Detailed summary of survey used in the study.

## Abbreviations

AUA: American Urological Association
DTC: direct-to-consumer
ED: erectile dysfunction
