# Supplementary material for: Individual Perceptions of the Efficacy, Quality, and Safety of Sexual Health Care Delivered via a Direct-to-Consumer Telehealth Platform: Retrospective Analysis of Data From a Cross-Sectional Survey Study
Source: JMIR Form Res. 2026 Sep 15;10:e95561. doi: 10.2196/95561 (PMC13577437; doi:10.2196/95561)
Supplement: Supplementary file 1 [file formative-v10-e95561-s001.docx]

SUPPLEMENTAL MATERIAL: SURVEY DETAILS

The original survey developed by the Hims & Hers Customer Insights and User Experience Research team comprised 22 items that queried individuals about their experience with Hims & Hers across a variety of safety and quality related areas. The first three items were used to confirm that individuals were current Hims & Hers customers at the time of survey completion. One item invited open-ended responses. Several other items asked individuals about specific products and features available through the platform, their opinions about future products that may be available through the platform, or their experiences with other digital health platforms. These questions were not relevant to the current study and therefore excluded from the analysis. The remaining items included:

| ***To what extent do you agree with the following statements about your experience with the telehealth platform?***  *Please respond on a scale of 1 to 5, 1 being “strongly disagree” and 5 being “strongly agree.”*   A. I fully understood the risks and benefits of the medication. B. I was confident my prescription was safe and appropriate for me. C. Side effects were clearly presented during the intake process. D. The entire process–from intake to prescription–felt medically safe. E. I trust Hims & Hers to offer safe and effective care. F. I believe Hims & Hers follows the proper rules and safety standards for healthcare. G. I felt the provider asked enough questions to fully understand my health history. |
| --- |
|  |
|  |
|  |
|  |
|  |
|  |
| ***How aware are you of the following safety practices used by Hims & Hers?***  *Please respond on a scale of 1 to 5, 1 being “not at all aware” and 5 being “very aware.”*   A. Medications sourced from FDA-regulated facilities. B. Licensed pharmacists formulating compounded treatments. C. Pharmacy safety protocols (e.g., clean rooms, sterility checks). D. Ongoing provider training and chart review. E. 24/7 access to licensed providers for safety follow-up and questions. |
|  |
|  |
|  |
|  |
|  |
| ***To what extent do you agree with the following statements based on this information?***  *Please respond on a scale of 1 to 5, 1 being “strongly disagree” and 5 being “strongly agree.”*   A. Knowing this information increases my trust in Hims & Hers. B. I feel confident in the safety of my treatment and care from Hims & Hers. |
|  |
|  |
| ***When you first became a customer you filled out an intake form as part of that process. Thinking back to that initial experience, how would you rate the intake experience with Hims & Hers?***  *Please respond on a scale of 1 to 5, 1 being “strongly disagree” and 5 being “strongly agree.”*   A. It was clinically thorough. B. It felt easy to complete. C. It clearly explained my treatment options. D. It helped me understand possible side effects. E. It made me feel more confident in starting treatment. F. It felt similar to the types of questions a provider might ask during an in-person appointment. G. It offered a more efficient and less stressful experience than an in-person visit. |
|  |
|  |
|  |
|  |
|  |
|  |
|  |
|  |
|  |
|  |
|  |
|  |
|  |
|  |
| ***Compared to traditional in-person healthcare, how would you rate the overall quality of your Hims & Hers care?***   - Much better - Somewhat better - About the same - Somewhat worse - Much worse |
| ***Compared to traditional in-person healthcare you’ve used, how would you rate your experience with Hims & Hers in the following areas?***  *Please respond on a scale of 1 to 5, 1 being “much worse” and 5 being “much better.”*   A. Ease of getting started / intake process. B. Thoroughness of questions that I was asked during the intake process. C. Clarity of treatment options and information. D. How well the medical provider listened and responded to my concerns. E. Personalization of my treatment plan. F. Transparency about side effects and risks. G. Privacy and discretion of the experience. H. Ease of communication with medical providers. I. Speed of access to care. J. Follow-up support and access to care after starting treatment. K. Overall sense of safety and medical quality. L. Trust in the care model and medical providers. M. Convenience of managing my care (e.g., subscriptions, changes) |
|  |
|  |
|  |
|  |
|  |
|  |
|  |
|  |
|  |
|  |
|  |
|  |
|  |
